# Antibacterial, In Vitro Anti-Inflammatory and Anti-Acne Activities of *Patchouli* Essential Oil

**DOI:** 10.3390/ph19060886

**Published:** 2026-06-02

**Authors:** Jiabao Cui, Hui Yang, Chenglin Wang, Lingyi Liu, Xiangxiang Zhu, Zhiqiang Wang, Shanshan Xiao, Dongbao Hu

**Affiliations:** 1 Food Science and Engineering Faculty, Kunming University of Science and Technology, Kunming 650500, China; 15045227277@163.com (J.C.); 13595862451@163.com (H.Y.); 18214601560@163.com (L.L.); 18208811248@163.com (X.Z.); 15798856140@163.com (Z.W.); 2 College of Modern Agricultural Engineering, Kunming University of Science and Technology, Kunming 650500, China; wangcl86@kust.edu.cn; 3 Department of Chemistry and Environmental Engineering, Yuxi Normal University, Yuxi 653100, China

**Keywords:** *Patchouli* essential oil, anti-acne, antibacterial, anti-inflammatory

## Abstract

**Objectives:** The study aimed to investigate the antibacterial, in vitro anti-inflammatory and anti-acne properties of *Patchouli* essential oil (PEO). **Methods**: PEO components were quantified by gas chromatography–mass spectrometry (GC-MS). The antibacterial activity of PEO against *Cutibacterium acnes* (*C. acnes*) and *Staphylococcus epidermidis* (*S. epidermidis*) was evaluated *via* MIC detection and growth curve analysis. Bacterial membrane integrity was assessed by detecting intracellular substance leakage. LPS-induced RAW264.7 cells inflammatory models and heat-inactivated *C. acnes*-stimulated human epidermal keratinocytes (HaCaT) cells acne models were constructed to explore the in vitro anti-inflammatory and anti-acne activities of PEO by measuring the secretion levels of pro-inflammatory cytokines. **Results:** PEO primarily contained α-guaiene, patchouli alcohol and α-bulnesene. It showed potent antibacterial activity with an MIC of 0.50 mg/mL, inhibiting bacterial growth and disrupting cell membrane integrity. PEO significantly alleviated cell damage and reduced the secretion of TNF-α, IL-1β and IL-6 in two inflammatory cell models, and suppressed excessive keratinocyte proliferation. **Conclusions:** PEO exerts anti-acne effects via dual antibacterial and anti-inflammatory pathways. This work provides valid experimental evidence for the application of PEO as a novel natural anti-acne agent.

## 1. Introduction

Acne is a chronic inflammatory skin disease involving the structure of hair follicle sebaceous glands. Its typical clinical manifestations are acne, papules, pustules, nodules, cysts and other forms of skin lesions. Acne tends to show up in areas where sebaceous glands are more abundant—most commonly the face, chest, and back [1,2,3]. Globally, the annual incidence of acne in 2024 was 20.50%. The highest rate, 28.3%, was seen in adolescents and young adults between 16 and 24 years old, while the rate stood at 19.3% in the 25–39 age group [4]. Although acne is not life-threatening, it causes no small amount of distress for patients, affecting not only their physical appearance but also their mental well-being [5]. For these reasons, there is a real need to find safe and effective alternative treatment options, and such efforts hold considerable promise.

The causes of acne are complex. Abnormal androgens can stimulate the sebaceous glands to secrete a large amount of oil, and the proliferation and metabolism of epidermal keratinocytes are unbalanced. In addition, the pathogens in the hair follicles are constantly attached and reproduced, which eventually leads to skin inflammation [6]. Among these, *Cutibacterium acnes* (*C. acnes*) can synthesize a biofilm, which is firmly attached to the hair follicle to survive and reproduce. This kind of survival mode will weaken the efficacy of antibiotics and make local inflammation unable to subside for a long time [7].

Meanwhile, existing studies have confirmed that there is a close relationship between *Staphylococcus epidermidis* (*S. epidermidis*) and acne. After the flora adheres to the skin surface, it is easy to cause local infection, which in turn triggers inflammation and discomfort [8].

In addition to the imbalance of flora, the metabolic disorder of keratinocytes in the sebaceous gland ducts of hair follicles is also an important core cause of acne. When the body has various abnormalities such as increased androgen secretion, excessive accumulation of skin sebum, and stimulation of local inflammatory factors, keratinocytes at the opening of hair follicles will have metabolic abnormalities, which are manifested as excessive proliferation, disordered differentiation and normal shedding mechanism. When keratinocytes undergo impaired desquamation, they accumulate together with sebum and bacterial metabolites, obstruct the pilosebaceous duct, and ultimately form microcomedones [9]. This pathological environment not only creates anaerobic conditions suitable for the mass reproduction of *C. acnes* but also helps the bacteria to continue to colonize and proliferate in the hair follicles, and gradually induces various acne skin lesions, eventually forming typical skin diseases such as inflammatory papules, pustules and nodules.

At the present stage, for the infection of *C. acnes*, erythromycin, clindamycin and other antibiotics are mostly used for intervention treatment [10,11]. However, from the perspective of practical application, traditional antibiotic therapy has certain limitations. Most patients will have different degrees of skin side effects after medication. The common manifestations are dry and red skin, local irritation and discomfort, and some patients will also have skin pigmentation and other problems. Furthermore, it may facilitate the development of bacterial resistance to antibiotics [12]. Essential oils derived from medicinal plants, take tea tree oil (TTO) and *Lavender* oil (LEO), for example. Both have been used to manage acne, thanks to their strong ability to kill bacteria and reduce inflammation [13,14]. Based on our research group’s previous gas chromatography–mass spectrometry (GC-MS) detection for chemical constituents of *Patchouli* essential oil (PEO), the major constituents were identified as α-guaiene (20.54%), patchouli alcohol (18.94%), α-bulnesene (16.29%), seychellene (10.41%), β-patchoulene (8.53%), and α-patchoulene (6.85%). Previous studies have shown that PEO, which is rich in α-guaiene, patchouli alcohol, and α-bulnesene, exhibits significant antibacterial activity against *Escherichia coli* (*E. coli*) and *Staphylococcus aureus* (*S. aureus*). Furthermore, PEO shows good anti-inflammatory activity in mouse models where inflammation is triggered by 12-O-tetradecanoylphorbol-13-acetate (TPA). In those experiments, it eased inflammation by lowering the expression of two key molecules—nitric oxide synthase (NOS) and interleukin-6 (IL-6)—in mouse macrophages. These earlier observations give us a helpful starting point for digging deeper into whether PEO might work against acne [15].

Based on the previous research results, this study explored and evaluated the inhibitory effect and mechanism of PEO on the main pathogenic strains of acne (*C. acnes* and *S. epidermidis*). At the same time, the anti-inflammatory activity of the substance in vitro was further verified by constructing a lipopolysaccharide (LPS)-induced RAW264.7 cells macrophage inflammation model.

Furthermore, an acne model was established using heat-killed *C. acnes*-induced human epidermal keratinocytes (HaCaT) cells to explore the anti-acne activity of PEO. This research aims to elucidate the anti-acne potential of PEO from antibacterial activity, anti-inflammatory effects and keratinocyte hyperproliferation inhibition perspectives, thus providing new data support for developing novel anti-acne agents.

## 2. Results and Discussion

### 2.1. Chemical Composition of Patchouli Essential Oil (PEO)

**Figure A1:**
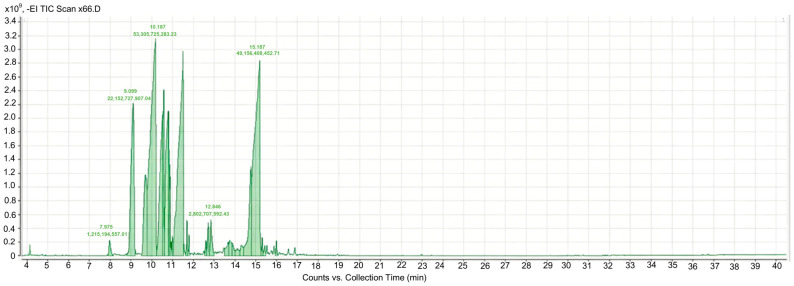
GC-MS chromatogram of *Patchouli* essential oil (PEO).

In this study, gas chromatography–mass spectrometry (GC-MS) was used to analyze and identify the volatile components of PEO. The specific test results are summarized in Table 1 and Figure A1. A total of 17 chemical components were successfully identified in this experiment, and their total relative content accounted for 99.04% of the total volatile oil components. The major constituents were α-guaiene (20.54%), patchouli alcohol (18.94%), α-bulnesene (16.29%), seychellene (10.41%), β-patchoulene (8.53%), and α-patchoulene (6.85%).

As an important active component in PEO, patchouli alcohol has been confirmed by previous studies to possess significant anti-inflammatory and antibacterial activities [16], This result is highly consistent with the core research purpose of this study to explore the antibacterial and anti-inflammatory biological activity of PEO. Related studies have confirmed that α-guaiene is one of the main volatile active components of PEO. Exerts remarkable bacteriostatic activity as well as antiplatelet aggregation properties [17,18], and it can be speculated that it is the key active substance of PEO to exert antibacterial pharmacological effects, whose specific mechanism of action remains to be further explored. Existing literature studies have shown that PEO with a high content of patchouli alcohol has excellent antibacterial ability against *S. aureus*, and the minimum inhibitory concentration of this essential oil against this strain can reach 2 mg/mL [19], which is consistent with the antibacterial experimental results of this study, further confirming the reliability of the biological activity of PEO and providing strong support for the conclusions of this study.

### 2.2. Antibacterial Activity and Mechanism of Patchouli Essential Oil (PEO)

#### 2.2.1. Minimum Growth Inhibitory Concentration (MIC)

The results of this experiment showed that PEO had a good inhibitory effect on *C. acnes* and *S. epidermidis*, and the minimum inhibitory concentration (MIC) of both was 0.50 mg/mL. In comparison, the MIC of TTO used as the positive control was 1 mg/mL. These findings indicate that PEO has a lower MIC and exhibits stronger antibacterial activity than TTO, suggesting its greater anti-acne potential. TTO is a well-established natural antibacterial agent with widely documented antimicrobial activity. In their study, Pekacar et al. showed that organic TTO compliant with European Pharmacopoeia specifications exerted remarkable antibacterial effects against both *C. acnes* and *S. epidermidis* [19]. Therefore, we further investigated the antibacterial action and underlying mechanism of PEO by evaluating the microbial growth curve, maximum growth rate (*µ*_max_), time to reach the maximum growth rate, as well as the leakage of UV-absorbing substances.

#### 2.2.2. Antibacterial Effect of *Patchouli* Essential Oil (PEO) on Growth Curves of *C. acnes* and *S. epidermidis*

**Figure 1:**
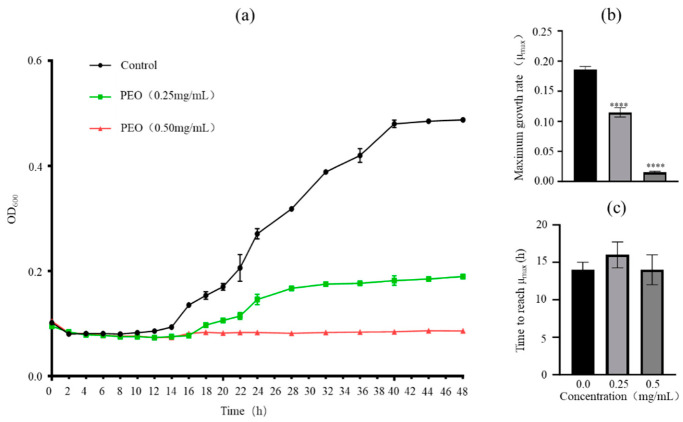
Effects of PEO on the growth profiles of *C. acnes*. (**a**) Growth curves of *C. acnes* in the presence of different concentrations of PEO (OD_600_ nm). (**b**) Maximum growth rate (*µ*_max_) of *C. acnes*. (**c**) Time to reach the *µ*_max_ of *C. acnes*. All data were shown as mean ± SD from three independent replicates (*n* = 3). Statistical significance was marked with asterisks: **** *p* < 0.0001, all compared with the Control group.

**Figure 2:**
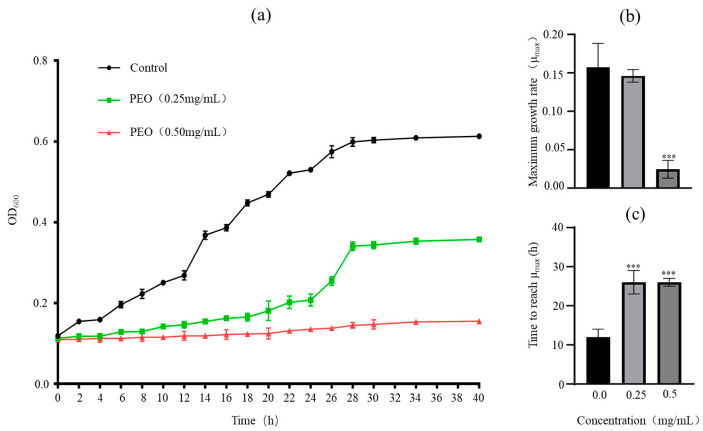
Effects of PEO on the growth profiles of *S. epidermidis*. (**a**) Growth curves of *S. epidermidis* in the presence of different concentrations of PEO (OD_600_ nm). (**b**) Maximum growth rate (*µ*_max_) of *S. epidermidis*. (**c**) Time to reach the *µ*_max_ of *S. epidermidis*. All data were shown as mean ± SD from three independent replicates (*n* = 3). Statistical significance was marked with asterisks: *** *p* < 0.001, all compared with the Control group.

In this study, microbial growth curves were determined to evaluate the antibacterial activity of PEO at concentrations of 0.25 mg/mL and 0.50 mg/mL against *C. acnes.* and *S. epidermidis*. The growth curve of *C. acnes* is presented in Figure 1a. The blank control group exhibited a typical S-shaped growth curve [20], whereas the growth and proliferation of *C. acnes* were markedly inhibited after PEO treatment. Correspondingly, the maximum specific growth rate (*µ_max_*) of *C. acnes* was significantly decreased (Figure 1b), and the time required to reach *µ*_max_ was remarkably prolonged following PEO intervention (Figure 1c). Consistent antibacterial effects were also observed in *S. epidermidis* (Figure 2a). Treatment with different concentrations of PEO effectively suppressed bacterial proliferation, reduced the *µ_max_* value (Figure 2b), and delayed the time to reach the maximum growth rate (Figure 2c). Collectively, these results indicate that PEO can significantly inhibit the growth and proliferation of the two common skin pathogenic bacteria in a concentration-dependent manner, with enhanced inhibitory effects observed as the PEO concentration increased. This kind of action law is not unique to PEO, and similar conclusions have also appeared in the existing research on various plant essential oils. Haman et al.’s study found that low doses of *Picea abies* essential oil can significantly prolong the lag phase of *E. coli*, which can increase its lag time from 14.1 h to 33.7 h, and this concentration condition will not have a significant effect on the *µ*_max_ of *E. coli* [21]. Our results, combined with earlier reports, indicate that PEO exerts its antibacterial effects largely by disrupting the growth dynamics of acne-related pathogens—a mechanism that closely resembles the principle action of most plant-based essential oils.

Combined with the data of bacterial growth kinetics, it can be seen that PEO can interfere with the normal growth cycle of pathogenic bacteria, greatly delay the time of bacteria entering the logarithmic growth phase, effectively inhibit the proliferation efficiency of strains, and down-regulate the *µ*_max_ [22]. The experimental results fully confirm that PEO has favorable activity against acne-causing bacteria and potential anti-acne effects, providing a reliable experimental basis for further exploration of its antibacterial mechanism and in vitro anti-acne activity.

#### 2.2.3. Evaluation of the Damage of Cell Membrane Integrity by *Patchouli* Essential Oil (PEO)

When the cell membrane structure of bacteria is damaged, the intracellular macromolecules such as nucleic acids and proteins will leak out, thus destroying the normal physiological and metabolic functions of bacteria. Nucleic acids and proteins have characteristic ultraviolet absorption at 260 nm and 280 nm wavelengths, respectively, which can be used to determine the damage of cell membrane [23,24]. Under normal conditions, biological macromolecules are difficult to penetrate the cell membrane, and the damage of the membrane structure will cause the leakage of intracellular components, so that the absorbance at 260 nm of the supernatant is increased. In this study, *C. acnes* and *S. epidermidis* in logarithmic growth phase were selected and cultured with PEO at half inhibitory concentration and minimum inhibitory concentration, respectively. To investigate the extent of cell membrane disruption in the tested bacterial strains by different concentrations of PEO, Log-phase *C. acnes* and *S. epidermidis* were cultured with PEO at half MIC and MIC. OD_260_ and OD_280_ were detected every 2 h to assess substance leakage.

**Figure 3:**
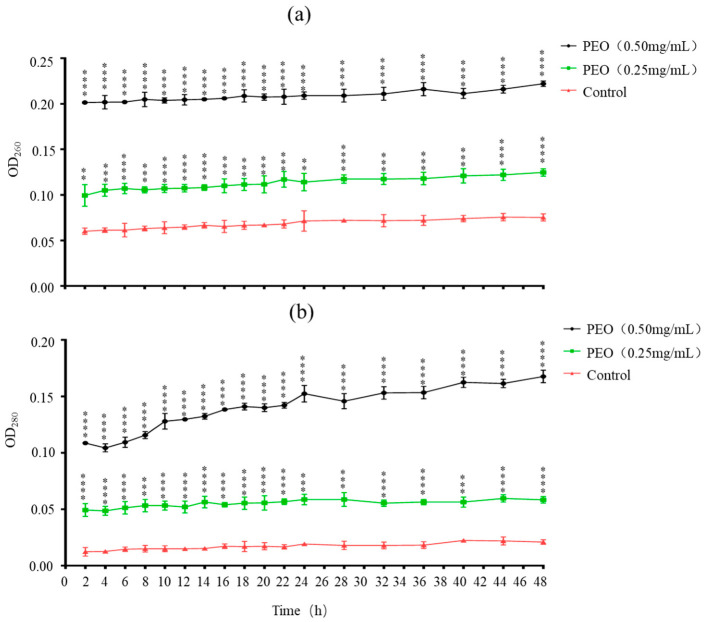
PEO impacts on intracellular UV-absorbing substance leakage. (**a**) Nucleic acid leakage at 260 nm after PEO treatment. (**b**) Protein leakage at 280 nm after PEO treatment. All data were shown as mean ± SD from three independent replicates (*n* = 3). Statistical significance was marked with asterisks: ** *p* < 0.01, *** *p* < 0.001, **** *p* < 0.0001, all compared with the Control group.

Figure 3 shows the UV absorption changes in *C. acnes* at OD_260_ and OD_280_ wavelengths under the action of PEO. The absorbance of the supernatant in the blank group remained basically stable, and the two values increased significantly after PEO intervention, confirming that PEO could damage the cell membrane. As the drug concentration went up, so did the absorbance values, which tells us that the extent of cell membrane damage correlates positively with the drug concentration. In summary, PEO can disrupt the cell membrane structure of *C. acnes*, increase membrane permeability, and thereby promote the leakage of intracellular macromolecules.

**Figure 4:**
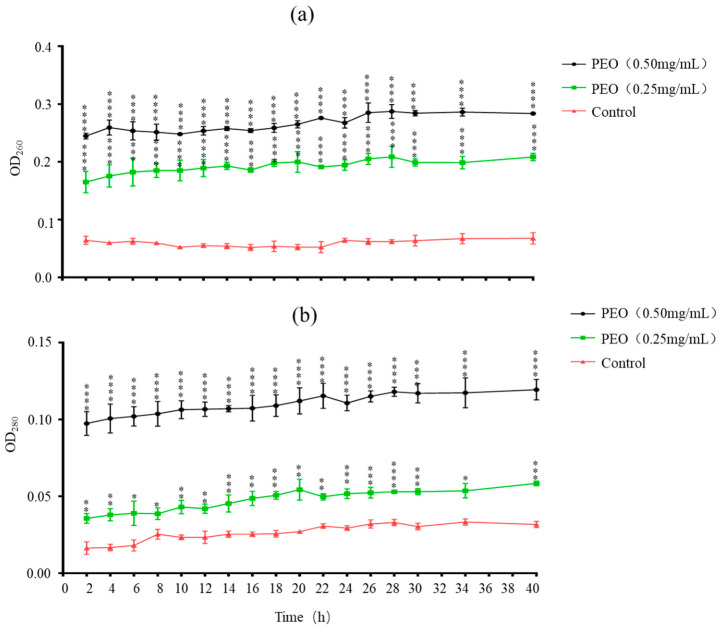
PEO impacts on intracellular UV-absorbing substance leakage. (**a**) Nucleic acid leakage at 260 nm following PEO treatment. (**b**) Protein leakage at 280 nm following PEO treatment. All data were shown as mean ± SD from three independent replicates (*n* = 3). Statistical significance was marked with asterisks: * *p* < 0.05, ** *p* < 0.01, *** *p* < 0.001, **** *p* < 0.0001, all compared with the Control group.

Similar results were also observed in *S. epidermidis* after PEO treatment. Compared with the blank control group, PEO damaged the bacterial cell membrane and gradually increased the absorbance of the bacterial supernatant (Figure 4). Consistent with the present findings, numerous plant essential oils have been reported to exert similar antibacterial effects. He et al. demonstrated that linalool could disrupt bacterial cell membranes by inducing oxidative stress, thereby causing the leakage of intracellular substances [25]. Similarly, *bergamot* essential oil destroys the bacterial membrane structure and increases membrane permeability, resulting in the release of intracellular nucleic acids and proteins [26]. Therefore, cell membrane damage is speculated to be one of the primary antibacterial mechanisms of PEO.

Nevertheless, the present study has certain limitations. This work only investigated the antibacterial activity of PEO, while its antibiofilm ability was not explored. It has been well documented that biofilm formation is closely associated with the occurrence and progression of acne lesions [27]. Therefore, further studies will be conducted to explore the antibiofilm effect and underlying mechanism of PEO, and in vivo experiments will be performed to verify its therapeutic efficacy. Thus, increased membrane permeability may be one important antibacterial pathway. Subsequent investigations will employ membrane potential analysis, transmission electron microscopy and enzymatic activity measurements to comprehensively clarify the mechanism and strengthen the robustness of our findings.

### 2.3. In Vitro Anti-Inflammatory Properties of Patchouli Essential Oil (PEO)

#### 2.3.1. Cell Counting Kit-8 (CCK-8)

**Figure 5:**
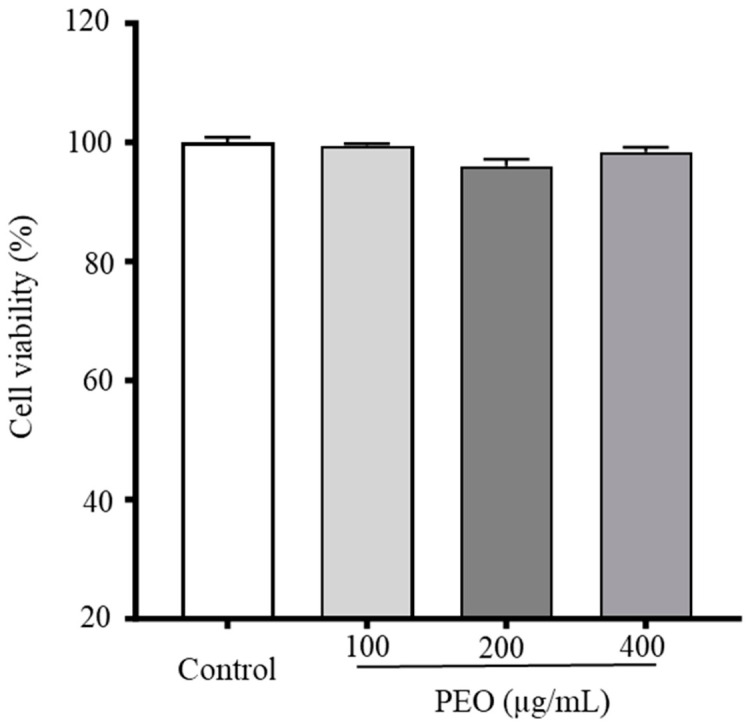
RAW264.7 cells treated with PEO were determined by the Cell Counting Kit-8 assay. All data were shown as mean ± SD from three independent replicates (*n* = 3).

PEO cytotoxicity toward RAW264.7 cells was tested at 100, 200, 400 µg/mL. As shown in Figure 5, when the concentration of PEO is in the range of 0 to 400 µg/mL, Compared with controls, PEO showed no obvious inhibition on cell viability. Based on the above results, PEO at 12.5, 25 and 50 µg/mL was determined for subsequent in vitro anti-inflammatory experimental analysis.

According to the data of cytotoxicity experiments, PEO at 400 µg/mL and below had no significant toxic and side effects on cells. Combined with preliminary experimental findings, low concentrations of 12.5, 25 and 50 µg/mL exerted prominent anti-inflammatory activity. Accordingly, these three non-cytotoxic doses were selected for all subsequent experiments in this study [28].

#### 2.3.2. LPS-Induced Inflammation in RAW264.7 Cells

**Figure 6:**
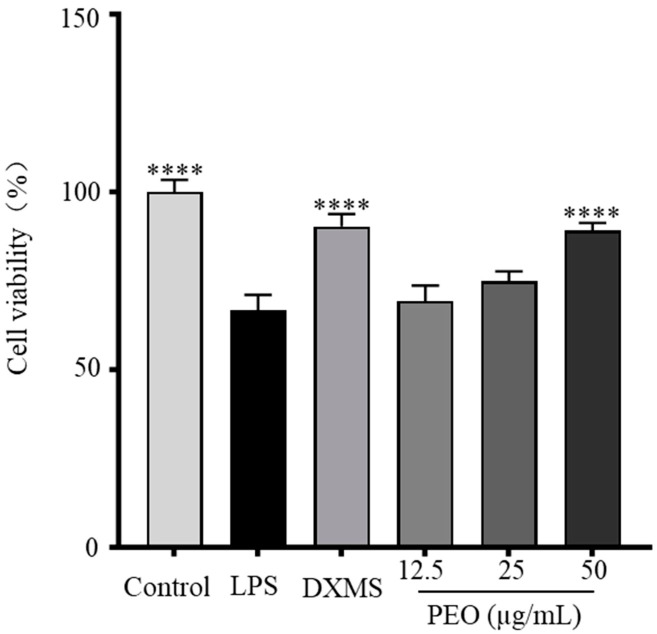
PEO alleviates LPS-induced damage in RAW264.7 cells macrophages. All data were shown as mean ± SD from three independent replicates (*n* = 3). Statistical significance was marked with asterisks: **** *p* < 0.0001, all compared with the LPS-treated group.

We used LPS to stimulate RAW264.7 cells to set up an inflammation model, and then ran CCK-8 assays to see whether PEO could protect against LPS-induced cell damage. As shown in Figure 6, the cell viability of the model group decreased significantly to 66.81 ± 4.36% (*p* < 0.0001), indicating that LPS stimulation successfully induced inflammatory injury in RAW264.7 cells, which was consistent with previous studies [29,30]. Compared with the model group, PEO treatment effectively restored cell viability in a dose-dependent manner. The cell viability was 69.37 ± 4.32% in the 12.5 µg/mL PEO group, 74.93 ± 2.70% in the 25 µg/mL group, and 89.18 ± 2.18% in the 50 µg/mL group. Low and medium concentrations of PEO exhibited limited protective effects, while 50 µg/mL PEO markedly alleviated LPS-induced cell damage, with a protective effect comparable to that of the positive control dexamethasone (DXMS, 1 µg/mL). These results demonstrated that PEO exerts a prominent protective effect against LPS-mediated inflammatory injury in RAW264.7 cells. Consistent with the present findings, previous studies have reported that plant essential oils can relieve LPS-induced cellular damage in a concentration-dependent manner [31,32].

**Figure 7:**
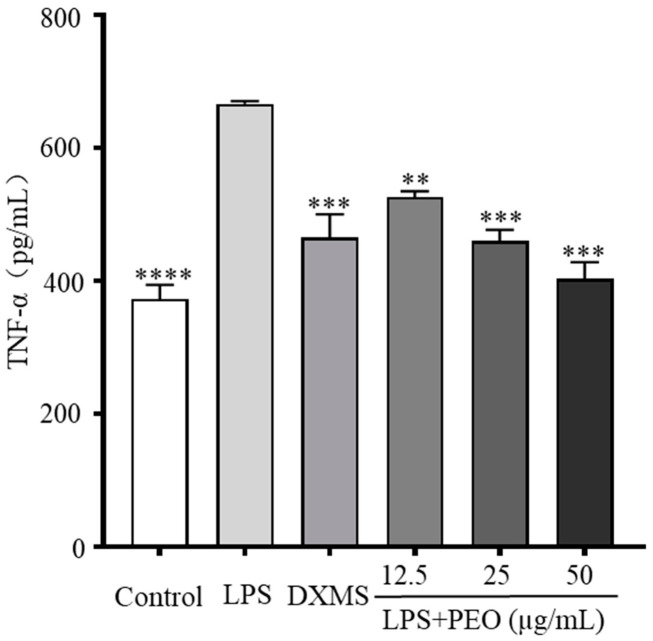
TNF-α secretion levels measured in this experiment. Data are presented as mean ± SD, based on three independent replicates (*n* = 3). Asterisks indicate statistical significance relative to the LPS-treated group: ** *p* < 0.01, *** *p* < 0.001, **** *p* < 0.0001.

**Figure 8:**
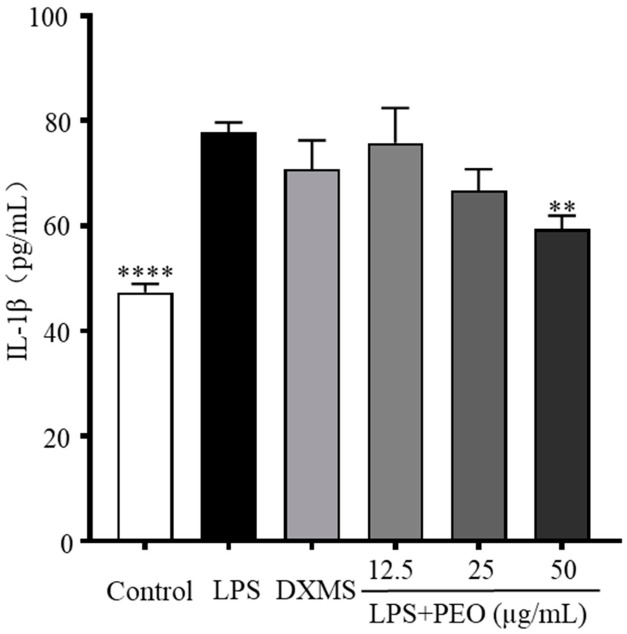
IL-1β secretion levels measured in this experiment. Data are presented as mean ± SD, based on three independent replicates (*n* = 3). Asterisks indicate statistical significance relative to the LPS-treated group: ** *p* < 0.01, **** *p* < 0.0001.

**Figure 9:**
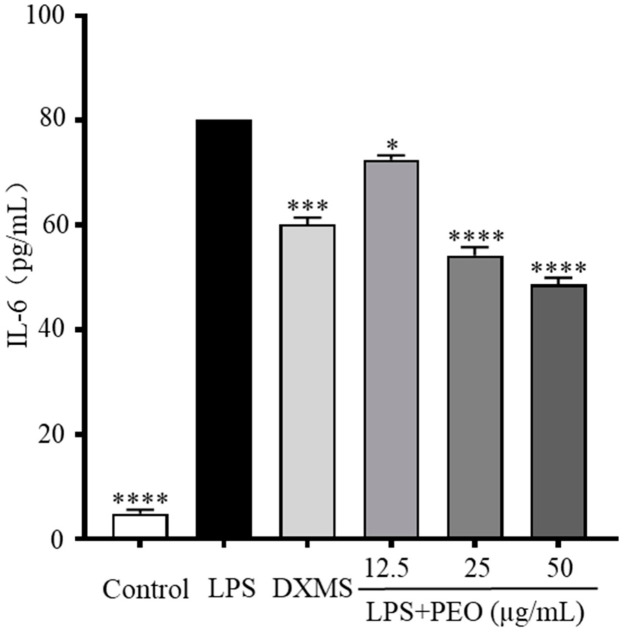
IL-6 secretion levels measured in this experiment. Data are presented as mean ± SD, based on three independent replicates (*n* = 3). Asterisks indicate statistical significance relative to the control group: * *p* < 0.05, *** *p* < 0.001, **** *p* < 0.0001.

In response to microbial infection and pathogenic stimulation, host cells release a series of inflammatory mediators, including nitric oxide (NO), prostaglandin E2 (PGE2), tumor necrosis factor-α (TNF-α), and interleukin-6 (IL-6), to initiate inflammatory responses [33]. Accordingly, the levels of inflammatory cytokines in the cell supernatant were determined by ELISA in this study. As presented in Figure 7, Figure 8 and Figure 9, the secretion of inflammatory cytokines was significantly upregulated in the LPS-stimulated model group, further confirming the successful establishment of the inflammatory model. All tested concentrations of PEO (12.5, 25, and 50 µg/mL) remarkably reduced the release of TNF-α and IL-6 (*p* < 0.01). However, the inhibitory effect on IL-1β was only observed in the 50 µg/mL PEO group, suggesting distinct anti-inflammatory responses of different cytokines toward PEO intervention [34].

Such differential inhibitory effects may be attributed to the different regulatory mechanisms underlying PEO-mediated anti-inflammatory activity. The NF-κB signaling pathway serves as a central regulator of the transcription and expression of TNF-α and IL-1β and is closely involved in the anti-inflammatory modulation of PEO [35,36,37]. Low and medium concentrations of PEO failed to fully block the activation of the NF-κB pathway, thereby showing a weak regulatory effect on IL-1β. In contrast, 50 µg/mL PEO significantly inhibited the phosphorylation of the NF-κB p65 subunit, which effectively suppressed the secretion of IL-1β [38]. Taken together, PEO shows clear anti-inflammatory properties in RAW264.7 cells macrophages exposed to LPS.

### 2.4. PEO Reduces C. acnes-Triggered Inflammation in a HaCaT Cell Acne Model

#### 2.4.1. Cell Counting Kit-8 (CCK-8)

HaCaT cells hyperproliferation as an indicator for evaluating anti-acne potential of test compounds in cell-based models has been widely reported in previous studies [39,40,41]. In the present study, we followed this established approach and used heat-killed *C. acnes*-induced abnormal cell proliferation as one of the parameters to assess the in vitro anti-acne potential.

**Figure 10:**
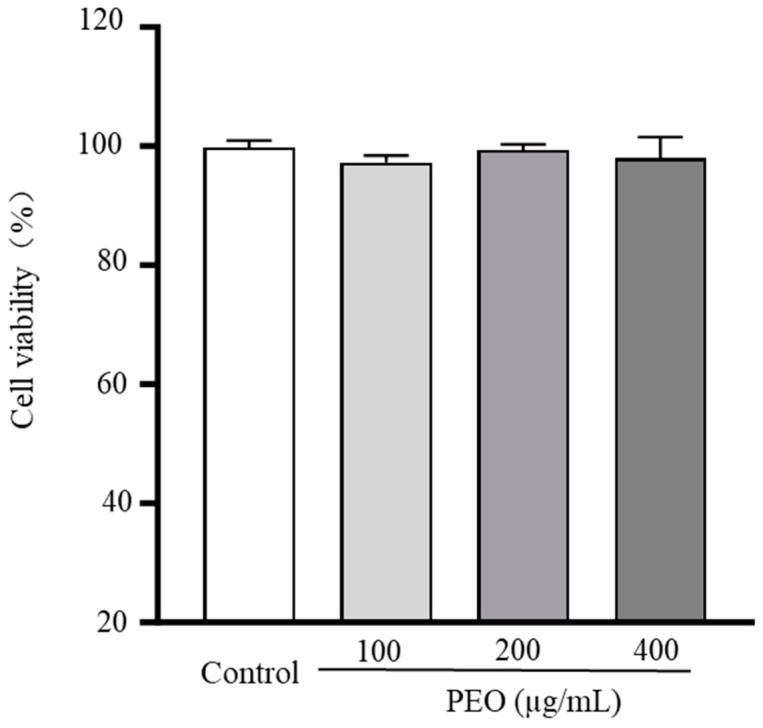
CCK-8 assay of HaCat cells following PEO treatment. All data were shown as mean ± SD from three independent replicates (*n* = 3).

Three concentrations of PEO (100, 200, and 400 µg/mL) were used to carry out cell experiments to explore the effect of this substance on the activity of HaCaT cells. It can be seen from the results of Figure 10 that when the PEO concentration was in the range of 0–400 µg/mL, it did not have a significant effect on the viability of HaCaT cells, and there was no significant difference between the test results of each group and the blank control group. According to the cytotoxicity evaluation, three concentrations of PEO (12.5, 25, and 50 µg/mL), all of which showed no cytotoxic effects on cells, were chosen for follow-up experiments.

Cytotoxicity assays revealed that PEO was not clearly toxic to cells at concentrations below 400 µg/mL. Preliminary experiments showed that 12.5, 25, and 50 µg/mL of PEO markedly inhibited cell inflammation triggered by heat-inactivated *C. acnes.* Hence, these three non-cytotoxic concentrations were adopted for all follow-up experiments.

#### 2.4.2. A HaCaT Cell Acne Model Induced by Heat-Killed *C. acnes*-Induced

**Figure 11:**
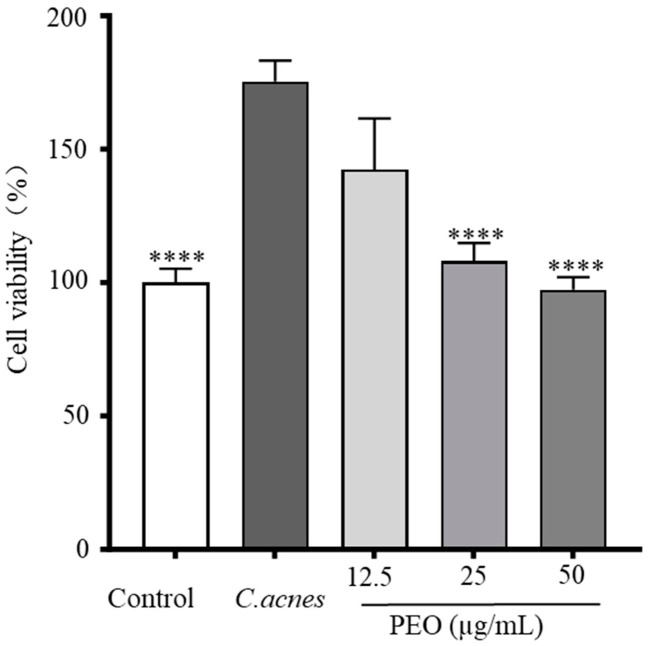
Cell viability measured by CCK-8 in an HaCaT cells model induced by heat-inactivated *C. acnes* and treated with PEO. Values are shown as mean ± SD from three separate experiments (*n* = 3). Significance versus the *C. acnes* group is denoted by asterisks: **** *p* < 0.0001.

From Figure 11, we can see that cell viability in the model group was much higher than in the control group. These results confirmed the successful establishment of the cellular acne model, which is consistent with previous studies [42,43]. Treatment with 25 µg/mL and 50 µg/mL PEO significantly inhibited the abnormal proliferation of HaCaT cells induced by heat-killed *C. acnes*, demonstrating a favorable in vitro anti-acne potential. On this basis, the regulatory effects of PEO on the release of inflammatory cytokines in the acne cell model were further determined using ELISA in this study.

#### 2.4.3. *Patchouli* Essential Oil (PEO) Reduces Pro-Inflammatory Cytokine Levels in *C. acnes*-Stimulated HaCaT Cells

**Figure 12:**
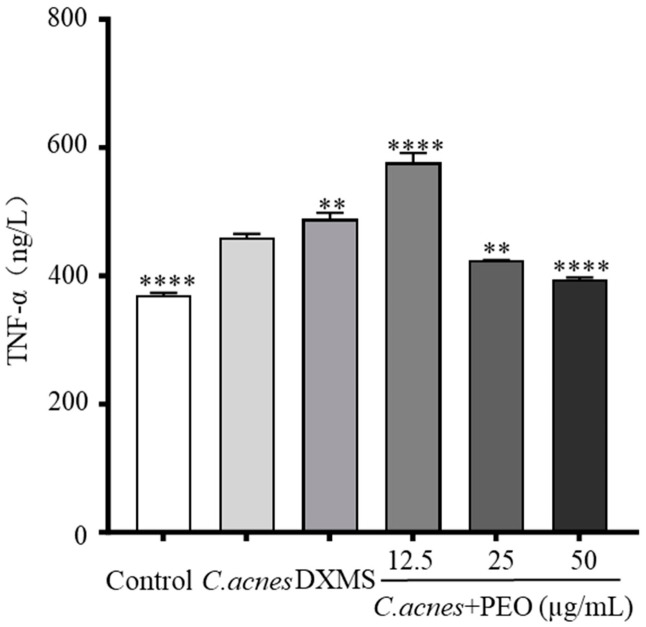
Concentrations of secreted TNF-α. Values are shown as mean ± SD from three separate experiments (*n* = 3). Significance versus the *C. acnes* group is denoted by asterisks: ** *p* < 0.01, **** *p* < 0.0001.

**Figure 13:**
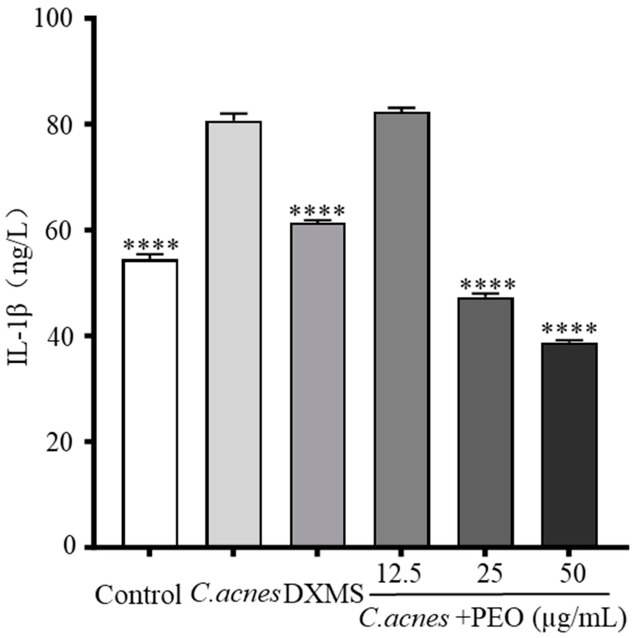
Concentrations of secreted IL-1β. Values are shown as mean ± SD from three separate experiments (*n* = 3). Significance versus the *C. acnes* group is denoted by asterisks: **** *p* < 0.0001.

**Figure 14:**
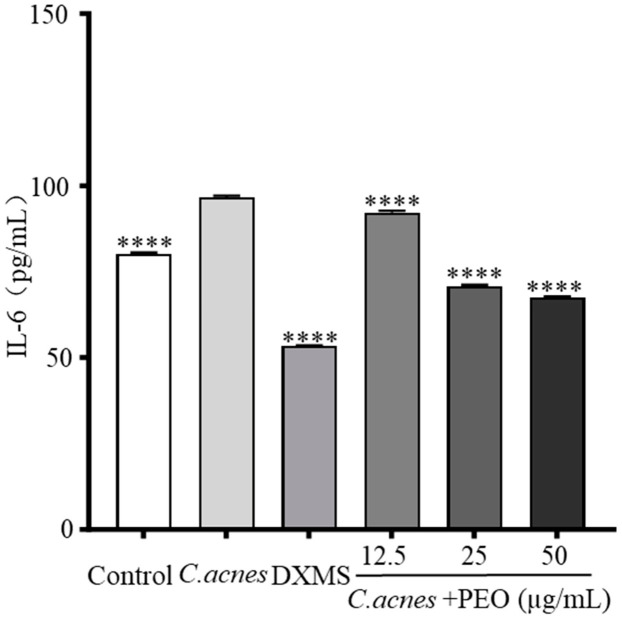
Concentrations of secreted IL-6. Values are shown as mean ± SD from three separate experiments (*n* = 3). Significance versus the *C. acnes* group is denoted by asterisks: **** *p* < 0.0001.

After stimulating HaCaT cells with heat-inactivated *C. acnes*, we harvested the supernatants and quantified TNF-α, IL-1β, and IL-6. Figure 12, Figure 13 and Figure 14 reveal that the model group secreted far more of these cytokines than the control group did (*p* < 0.0001), confirming that the *C. acnes*-induced HaCaT cells acne model was properly established.

Figure 12 shows that PEO at different concentrations had bidirectional effects on TNF-α secretion triggered by heat-inactivated *C. acnes*. The low dose (12.5 µg/mL) significantly boosted TNF-α secretion (*p* < 0.0001). The medium and high doses could significantly reduce the increase in TNF-α level induced by *C. acnes* (*p* < 0.01). This bidirectional regulation is likely due to the activation of NF-κB pathway, a central pathway that controls pro-inflammatory cytokine expression [43]. Mechanistically, the low PEO concentration may fail to block NF-κB activation, which in turn increases TNF-α secretion. The medium and high concentrations, however, effectively suppressed NF-κB activation and thereby limited TNF-α overproduction.

It can be seen from the results of Figure 13 that PEO can effectively inhibit the expression of IL-1β inflammatory factors induced by heat-inactivated, and the inhibitory effect shows obvious concentration-dependent characteristics. The low dose (12.5 µg/mL) had little effect on IL-1β levels, but the medium and high doses markedly reduced the *C. acnes* -induced rise in IL-1β (*p* < 0.0001), and the inhibition grew stronger with increasing concentration. This effect can be explained by the fact that PEO at high concentrations downregulates phosphorylation of the NF-κB p65 subunit, which in turn blocks IL-1β transcription and lowers its secretion [44].

Figure 14 shows that PEO treatment drove a concentration-dependent decline in IL-6 secretion. As PEO concentrations rose from 12.5 to 50 µg/mL, IL-6 expression dropped steadily, and at 50 µg/mL, IL-6 levels fell to near those of the control group. All PEO-treated groups differed significantly from the model group (*p* < 0.0001). This concentration-dependent inhibition operated mainly through two routes. First, PEO directly lowered IL-6 transcription via suppression of the NF-κB pathway [35]. Second, PEO indirectly reduced IL-6 secretion by boosting intracellular antioxidant enzyme activity and cutting down reactive oxygen species (ROS) production [45].

The above results indicated that PEO significantly reduced the secretion of inflammatory cytokines in the cellular acne model, exhibiting excellent anti-acne activity *in vitro*. Accumulated evidence has revealed that multiple plant essential oils, including those derived from *Angelica sinensis* [46] and *Schizonepeta tenuifolia* [47,48], can ameliorate inflammatory injuries in acne models by modulating the expression of core pro-inflammatory cytokines. Consistent with these findings, the present study preliminarily confirms the potent in vitro anti-acne efficacy of PEO.

## 3. Materials and Methods

### 3.1. Materials and Reagents

PEO and tea tree essential oil (TTO) used in this study were kindly provided by Dr. Dongbao Hu from Yuxi Normal University, which were prepared from dried *Patchouli* leaves by steam distillation. In summary, cleaned and pulverized leaves were transferred to a distillation apparatus and distilled with deionized water for 5 h. After distillation, the collected distillate was left to stand, allowing it to stratify. The upper oil phase was then separated. To remove residual moisture, anhydrous sodium sulfate was added. The mixture was subsequently filtered to obtain purified PEO. The resulting essential oil was sealed and stored at 4 °C in darkness until use in later experiments.

The following reagents were used. Cell culture materials—Dulbecco’s Modified Eagle’s Medium (DMEM), fetal bovine serum (FBS), penicillin/streptomycin—from GIBCO (Grand Island, NY, USA). Dimethyl sulfoxide (DMSO) and nutrient broth (NB) from Solarbio (Beijing, China). Cell Counting Kit-8 (CCK-8) from Abbkine (Wuhan, China). Gifu Anaerobic Medium (GAM) from HopeBio (Qingdao, China). For cytokine detection, enzyme-linked immunosorbent assay (ELISA) kits (SenBeiJia, Nanjing, China) were used, targeting tumor necrosis factor-alpha (TNF-α), interleukin-1β (IL-1β), and interleukin-6 (IL-6).

### 3.2. Bacterial Strains and Culturing

*C. acnes* (ATCC 6919) and *S. epidermidis* (ATCC 12228) were both acquired from Guangdong Microbial Culture Collection Center. The former was anaerobically incubated in GAM at 37 °C for 48 h under strict aseptic conditions, while the latter was aerobically cultured in NB medium at the same temperature for 24 h.

### 3.3. Cell Culture

We cultured two cell lines in this study: RAW264.7 cells (ATCC CLS 300493) and HaCaT cells (ATCC^®^ TIB-71™). Both were maintained in DMEM supplemented with 10% FBS and 1% penicillin-streptomycin, at 37 °C in a 5% CO_2_ atmosphere. The RAW264.7 cells came from the Kunming Institute of Zoology, Chinese Academy of Sciences (CAS), whereas the HaCaT cells were obtained from the Cell Bank of the Shanghai Institute of Biochemistry and Cell Biology, also part of CAS.

### 3.4. Gas Chromatography–Mass Spectrometry (GC-MS) Analysis

This protocol was revised based on previous reports by Xiao et al. [49]. GC-MS analysis was conducted via an Agilent 7890B gas chromatograph (Santa Clara, CA, USA) coupled with a LECO Pegasus BT mass spectrometer (LECO Corporation, St. Joseph, MI, USA), using a DB-5 capillary column (Agilent Technologies, Santa Clara, CA, USA). The oven was held at 45 °C for 2 min, ramped to 230 °C at 8 °C/min and maintained for 10 min. Helium flow was 1.0 mL/min, and injector temperature was 250 °C. Samples were injected after 3 min desorption at 250 °C. EI ionization was applied with optimized MS parameters within 33–450 amu mass range [50]. GC-FID detection was carried out on Shimadzu GC2010, with only detector temperature set to 230 °C, and all other conditions consistent with GC-MS.

### 3.5. Antimicrobial Activities

#### 3.5.1. The Minimum Inhibitory Concentration (MIC) of *Patchouli* Essential Oil (PEO) Was Determined

In this experiment, the minimum inhibitory concentration (MIC) of PEO was detected by 96-well plate gradient dilution method [51]. During the experiment, the PEO raw material was dissolved with dimethyl sulfoxide (DMSO), and then diluted with nutrient broth (NB) medium to obtain an initial concentration of 8 mg/mL. Serial twofold dilutions were subsequently prepared to obtain final concentrations of 4, 2, 1, 0.5, 0.25, and 0.125 mg/mL.

*C. acnes* and *S. epidermidis* stored at −80 °C were revived on NB agar plates and GAM agar plates, respectively, and incubated overnight at 37 °C (*C. acnes* was cultured in an anaerobic chamber for 48 h). The purified single colony was selected and placed in 0.85% sodium chloride solution to prepare the bacterial suspension, and then the turbidity of the bacterial solution was adjusted to 0.5 McBurney standard. At this time, the concentration of the bacterial solution was about 10^6^ CFU/mL.

For the MIC assay, 100 µL of bacterial suspension and 100 µL of PEO-containing medium at each concentration were added to the wells of a 96-well microplate. Cultured at 37 °C for 24 h, while *C. acnes* was anaerobically incubated for 48 h. The absorbance value at 600 nm wavelength was detected by microplate reader to determine the growth status of bacteria. All samples in this experiment were set up 6 groups of parallel tests, and TTO was selected as the positive control. MIC was defined as the lowest PEO concentration that could completely inhibit the visible growth of bacteria, and the final result unit was mg/mL.

#### 3.5.2. Growth Curve Determination

To evaluate how well PEO inhibits the growth of *C. acnes* and *S. epidermidis*, we used a spectrophotometric method. The procedure was based on previously described protocols [48], although we introduced a few minor adjustments to better suit our experimental setup. Briefly, *C. acnes* and *S. epidermidis* log-phase bacteria were harvested via centrifugation at 8000× *g* and 4 °C for 5 min and diluted with physiological saline to a concentration of 10^8^ CFU/mL. 2 mL bacterial suspension was added into flasks with 200 mL fresh sterile medium. After that, we added PEO to the culture medium so that the final concentrations reached 0.25 mg/mL and 0.50 mg/mL, respectively. As a control, we prepared cultures of *C. acnes* and *S. epidermidis* that contained 0.50% DMSO but no PEO. All cultures were placed on a shaker at 120 rpm and kept at 37 °C. To keep track of bacterial growth and changes in cell density, we measured the absorbance at 600 nm (OD_600_) every two hours using a UV-Vis spectrophotometer (Agilent Technologies, San Jose, CA, USA).

We calculated the growth rates (*µ*) of *C. acnes* and *S. epidermidis* under different PEO concentrations using the OD_600_ values we collected. The following formula was applied for this calculation [52,53]:

*µ* = (lnA_1_ − lnA_2_)/(t_1_ − t_2_) (1)

where A_1_ and A_2_ denote OD_600_ values at corresponding time points t_1_ and t_2_.

#### 3.5.3. Effect of *Patchouli* Essential Oil (PEO) Treatment on Cell Membrane Integrity

Follow-up experiments were carried out according to the above research methods [54]. The bacteria of *C. acnes* and *S. epidermidis* in the logarithmic growth phase were collected by centrifugation, and the fresh medium was replaced to re-suspend and adjust the cell density of the bacterial solution to 10^7^ CFU/mL. PEO reagent pre-dissolved by DMSO was added to the culture medium, and two final concentration groups of 0.50 mg/mL and 1.0 mg/mL were set up. At the same time, the pure culture medium containing 0.50% DMSO was used as a blank control. The samples in each group were cultured at 37 °C for 24 h, and then centrifuged at 8000× *g* and 4 °C for 5 min to complete the sample collection and processing. The resulting supernatants were collected, and the absorbance was measured at 260 nm and 280 nm using a UV-Vis spectrophotometer to quantify the leakage of nucleic acids and proteins from the cells. Higher absorbance values indicated a greater degree of cell membrane injury.

### 3.6. In Vitro Anti-Inflammatory Activities

#### 3.6.1. Cell Counting Kit-8 (CCK-8)

In this study, CCK-8 cell proliferation assay was used to analyze the toxic effect of PEO on RAW264.7 cells, and the cell viability under LPS-induced inflammatory injury was measured. In order to screen out the safe concentration of PEO without obvious damage to cells, this experiment set up gradient PEO concentrations of 100, 200, 400 µg/mL, and incubated with RAW264.7 cells for 24 h, respectively, to carry out cytotoxicity screening experiments. On this basis, cells were pretreated with the selected non-cytotoxic PEO concentrations, and then stimulated with LPS (1 µg/mL) to establish inflammatory cell model. Based on that, cells received a pretreatment with the selected non-cytotoxic PEO concentrations. Then LPS (1 µg/mL) was applied to induce inflammation, creating the inflammatory cell model. After treatment, 20 µL of CCK-8 reagent was added into each well. The plates were then left to incubate for another 4 h at 37 °C. Following that, absorbance at 450 nm was measured with a microplate reader. Cell viability was calculated as a percentage of the untreated control group, using the method described in reference [49].

#### 3.6.2. Assessing the Anti-Inflammatory Effect

Cells went into 96-well plates at 1 × 10^4^ per well and sat overnight to attach. Then we added 1 µg/mL LPS to trigger inflammation [55]. To test anti-inflammatory effects, we switched to serum-free medium containing LPS along with the essential oil at 12.5, 25, or 50 µg/mL. Incubation continued for another 24 h.

#### 3.6.3. Enzyme-Linked Immunosorbent Assay (ELISA) of Cytokines

To measure key inflammatory cytokines, we used the LPS-induced RAW264.7 cells model. TNF-α, IL-1β, and IL-6 in culture supernatants were quantified by ELISA. All steps followed the kit manufacturer’s instructions. For sample collection, the two cell models were separately treated with either LPS or heat-inactivated *C. acnes* for 24 h. After that, supernatants were collected and analyzed with the corresponding ELISA kits. Absorbance was read at 450 nm. Cytokine concentrations were then calculated from standard curves prepared for each assay.

### 3.7. In Vitro Anti-Acne Activity of Patchouli Essential Oil (PEO)

#### 3.7.1. Cell Counting Kit-8 (CCK-8)

PEO cytotoxicity and cell activity were determined by CCK-8 assay. To evaluate cell safety, HaCaT cells were exposed to PEO at 100, 200, and 400 µg/mL for 24 h, from which safe (non-toxic) concentrations were selected. For the anti-acne inflammation model that followed, HaCaT cells received a pretreatment with these screened safe PEO concentrations, and then stimulated with heat-killed *C. acnes* to establish cell acne model. After the cell culture of each group was completed, 20 µL CCK-8 reagent was added to each well system and incubated at 37 °C for 4 h. The optical density at 450 nm was quantified with a microplate spectrophotometer, and cell viability was subsequently determined and presented relative to the untreated blank control as a percentage [50].

#### 3.7.2. Establishment of a Cellular Acne Model Induced by Heat-Killed *C. acnes*

The method was adapted from Xiao et al. [50]. Following trypsinization, HaCaT cells in the logarithmic growth phase were selected and resuspended in fresh complete medium after centrifugation. The cells were seeded in a culture plate at a cell density of 5 × 10^4^ cells/well and then cultured in a constant temperature incubator at 37 °C and 5% CO_2_ for 24 h to make the cells fully adherent [56]. After the cells had firmly attached to the culture surface, we removed the original medium and replaced it with fresh medium containing heat-killed *C. acnes* at a final concentration of 50 µg/mL [57]. To build the cellular acne model, the cells were then incubated together with the heat-killed bacteria for a full 24 h [56].

#### 3.7.3. Cytokine Assay by ELISA

The production of pro-inflammatory cytokines was quantified in HaCaT cells challenged with heat-inactivated *C. acnes*. The concentrations of TNF-α, IL-1β and IL-6 in cell culture supernatants were assayed using ELISA kits in strict accordance with the manufacturer’s protocols. After treatment of the two cell models with either LPS or heat-killed *C. acnes* for 24 h, the supernatants were collected and assayed using the corresponding ELISA kits. Absorbance values were detected at 450 nm, and the concentrations of inflammatory cytokines were computed using calibration curves.

### 3.8. Statistical Analysis

All assays were conducted with three independent biological replicates, each containing three technical replicates. Results are shown as mean ± standard deviation (SD) derived from three separate experiments. The normality of data distribution and homogeneity of variances were confirmed before any statistical comparisons. For comparisons between two experimental groups, Student’s unpaired *t*-test was employed. For comparisons among three or more groups, one-way ANOVA was performed followed by Tukey’s multiple comparison test. Differences were considered statistically significant when *p* < 0.05. GraphPad Prism (version 8.0 or 9.0) was used for all statistical analyses.

## 4. Conclusions

In conclusion, this study investigated the antibacterial, anti-inflammatory, and anti-acne activities of PEO in vitro. The results demonstrated that PEO exhibits prominent antibacterial effects against *C. acnes* and *S. epidermidis*. It also effectively alleviated LPS-induced inflammatory injury in RAW264.7 cells macrophages and significantly ameliorated excessive cell proliferation and inflammatory responses in the in vitro acne cell model. Nevertheless, the present study was limited to in vitro validation. Further in vivo experiments and in-depth mechanistic studies are required to fully clarify the underlying molecular mechanisms and validate the clinical potential of PEO as a promising anti-acne agent.

## Figures and Tables

**Table 1:** Chemical components of *Patchouli* essential oil (PEO) by GC-MS analysis.

No.	Compound	Content (%)	Molecular Formula	CAS Number	RT (min)
1	β-Patchoulene	8.53	C_15_H_24_	514-51-2	9.099
2	Bicyclo[5.2.0]nonane, 4-ethenyl-4,8,8-trimethyl-2-methylene-	4.65	C_15_H_24_	242794-76-9	9.697
3	α-Guaiene	20.54	C_15_H_24_	3691-12-1	10.187
4	Seychellene	10.41	C_15_H_24_	20085-93-2	10.52
5	α-Patchoulene	6.85	C_15_H_24_	560-32-7	10.779
6	Valerena-4,7(11)-diene	1.87	C_15_H_24_	351222-66-7	10.818
7	γ-Patchoulene	1.09	C_15_H_24_	508-55-4	10.872
8	γ-Gurjunene	0.39	C_15_H_24_	22567-17-5	11
9	α-Bulnesene	16.29	C_15_H_24_	3691-11-0	11.504
10	nootkatene	0.29	C_15_H_22_	5090-61-9	11.788
11	(+)-Norpatchoulenol	0.69	C_14_H_22_O	41429-52-1	12.719
12	Costol	3.11	C_15_H_24_O	515-20-8	13.669
13	γ-himachalene	0.89	C_15_H_24_	1000140-08-0	14.1
14	(−)-Globulol	3.94	C_15_H_26_O	489-41-8	14.766
15	Patchouli alcohol	18.94	C_15_H_26_O	5986-55-0	15.187
16	α-Cyperone	0.31	C_15_H_22_O	473-08-5	15.305
17	Ceramides	0.25	C_16_H_28_O	1000333-80-8	15.996

## Data Availability

The original contributions presented in this study are included in the article. Further inquiries can be directed to the corresponding authors.

## Abbreviations

The following abbreviations are used in this manuscript:

PEO	*Patchouli* essential oil
GC-MS	Gas Chromatography–Mass Spectrometry
*C. acnes*	*Cutibacterium acnes*
*S. epidermidis*	*Staphylococcus epidermidis*
*S. aureus*	*Staphylococcus aureus*
*Escherichia coli*	*E. coli*
MIC	Minimum growth-inhibitory concentration
LPS	Lipopolysaccharide
TNF-α	Tumor necrosis factor-α
IL-1β	Interleukin-1β
IL-6	Interleukin-6
HaCaT	Human epidermal keratinocyte
CCK-8	Cell Counting Kit-8
TTO	Tea tree essential oil
LEO	*Lavender* essential oil
TPA	12-O-Tetradecanoylphorbol-13-acetate
NOS	nitric oxide synthase
DMEM	Dulbecco’s Modified Eagle’s Medium
FBS	Fetal Bovine Serum
DMSO	Dimethyl Sulfoxide
NB	Nutrient Broth
GAM	Gifu Anaerobic Medium
ELISA	Enzyme-Linked Immunosorbent Assay
SD	standard deviation
*µ*_max_	Maximum growth rate
NF-κB	Nuclear factor-κB
DXMS	Dexamethasone
NO	Nitric Oxide
PGE2	Prostaglandin E2
